# The role of hypercapnia in acute respiratory failure

**DOI:** 10.1186/s40635-019-0239-0

**Published:** 2019-07-25

**Authors:** Luis Morales-Quinteros, Marta Camprubí-Rimblas, Josep Bringué, Lieuwe D. Bos, Marcus J. Schultz, Antonio Artigas

**Affiliations:** 1 Intensive Care Unit, Hospital Universitario Sagrado Corazón, Carrer de Viladomat, 288, 08029 Barcelona, Spain; 2 Department of Medicine, Universitat Autònoma de Barcelona, Bellatera, Spain; 3 Critical Care Center, Corporació Sanitària I Universitària Parc Taulí, Sabadell, Spain; 4 Institut d’Investigació i Innovació Parc Taulí (I3PT), Sabadell, Spain; 5 Department of Intensive Care, Amsterdam University Medical Centers, University of Amsterdam, Amsterdam, The Netherlands; 6 Respiratory Medicine, Amsterdam University Medical Centers, University of Amsterdam, Amsterdam, The Netherlands; 7 Laboratory of Experimental Intensive Care and Anesthesiology (L·E·I·C·A), Amsterdam University Medical Centers, University of Amsterdam, Amsterdam, The Netherlands; 8 Mahidol Oxford Tropical Medicine Research Unit (MORU), Mahidol University, Bangkok, Thailand; 9 Centro de Investigación en Red de Enfermedades Respiratorias (CIBERES), Madrid, Spain

**Keywords:** Acute respiratory failure, Acute respiratory distress syndrome, Carbon dioxide, Permissive hypercapnia, Hypercapnia, Hypercapnic acidosis

## Abstract

The biological effects and physiological consequences of hypercapnia are increasingly understood. The literature on hypercapnia is confusing, and at times contradictory. On the one hand, it may have protective effects through attenuation of pulmonary inflammation and oxidative stress. On the other hand, it may also have deleterious effects through inhibition of alveolar wound repair, reabsorption of alveolar fluid, and alveolar cell proliferation. Besides, hypercapnia has meaningful effects on lung physiology such as airway resistance, lung oxygenation, diaphragm function, and pulmonary vascular tree.

In acute respiratory distress syndrome, lung-protective ventilation strategies using low tidal volume and low airway pressure are strongly advocated as these have strong potential to improve outcome. These strategies may come at a price of hypercapnia and hypercapnic acidosis. One approach is to accept it (permissive hypercapnia); another approach is to treat it through extracorporeal means. At present, it remains uncertain what the best approach is.

**Electronic supplementary material:**

The online version of this article (10.1186/s40635-019-0239-0) contains supplementary material, which is available to authorized users.

## Background

Patients with acute respiratory failure almost always develop gas exchange derangements that may result in hypercapnia [1]. Lung-protective ventilation strategies are strongly recommended to prevent additional lung injury [2, 3], but these strategies have a strong potential to increase plasma carbon dioxide levels further. One approach is to accept this, i.e., “permissive hypercapnia,” with the option to correct respiratory acidosis by slow bicarbonate infusion for blood buffering. Extracorporeal decapneization by utilizing “extracorporeal CO_2_ removal” (ECCO_2_R) is an appealing alternative for permissive hypercapnia but comes with the typical risks of extracorporeal circulation.

Hypercapnia has been suggested to have beneficial effects, including a reduction in pulmonary inflammation and alveolar oxidative stress [4–7]. Hypercapnia, however, may also have deleterious effects, such as impairments in tissue repair and decreased alveolar fluid clearance [8–11]. Seen these seemingly opposite effects, it becomes increasingly important to determine the net consequence of hypercapnia. Indeed, the number one question in patients with acute respiratory distress syndrome (ARDS), who either have hypercapnia or develop hypercapnia as a consequence of lung-protective ventilator settings, is whether hypercapnia should be accepted, or prevented and corrected.

This narrative review provides an overview of the various biological and physiological effects of hypercapnia and discusses current strategies affecting the plasma carbon dioxide levels in ARDS patients.

## Biological effects of hypercapnia—preclinical studies

Several preclinical studies have shown hypercapnia displays profound impact on alveolar cells and humoral factors that all could play a role in lung injury. Some of these effects can be seen as potentially beneficial, while others, in theory, could be harmful.

### Effects of hypercapnia on alveolar cells (Table 1)

Hypercapnia has been found to decrease microvascular permeability, lung edema formation, and bronchoalveolar lavage protein content in the rabbit lungs subjected to ex vivo ventilation with high pressures [4]. Hypercapnia also reduced histologically confirmed lung injury in ventilated mice [5]. The plausible mechanisms are related to the action of carbon dioxide upon the nuclear factor kappa pathway, which prevents p65 translocation and reducing inflammation [12, 13].

**Table 1:** Alveolar cellular effects of hypercapnia: summary of in vivo and ex vivo experiments on the effects of hypercapnia

Study	Experimental model	Applied CO_2_	Cellular effects
Broccard et al. [4]	VILI ex vivo (rabbit)	PaCO_2_ target 70–100 mmHg	HCA reduced microvascular permeability, lung edema formation, and BAL protein content
Yang et al. [20]	VILI in vivo (rat) and in vitro alveolar epithelial cells	PaCO_2_ target 80–100 mmHg	HCA attenuated microvascular leak, oxidative stress, and inflammation
Doerr et al. [65]	VILI/plasma wound resealing. Ex vivo (rat) and in vitro alveolar epithelial cell	12%	Hypercapnia reduced plasma membrane resealing in vivo and in vitro
O’Toole et al. [8]	In vitro three cell respiratory lines	10, 15%	Hypercapnia reduced rate of wound closure (cell migration) via NF-κB pathway inhibition
O’Croinin et al. [17]	*E. coli* pneumonia (48 h). In vivo (rat)	8%	Hypercapnia worsened lung injury induced by prolonged untreated *E. coli* pneumonia
Wang et al. [21]	Endotoxin stimulation. In vitro human and mouse macrophages	5, 9, 12.5, 20%	Hypercapnia inhibited macrophage phagocytosis

*HCA* hypercapnic acidosis, *VILI* ventilator-induced lung injury, *BAL* bronchoalveolar lavage, *NF-kB* nuclear factor kappa B

It also attenuated apoptosis in rabbits subjected to ischemia and reperfusion injury [6], and buffering hypercapnic acidosis worsened lung injury in this model [14]. One of the proposed mechanisms is the inhibitory action of CO_2_ on the ADAM-17 (a sheddase), thus preventing the activation of the p44/p42 MAPK pathway and, by this way, reducing apoptosis [15]. Finally, hypercapnia resulted in less cell injury and neutrophil adherence to endothelial monolayers in stimulated pulmonary endothelial cells [16].

Contrasting, in rats exposed to hypercapnia for 48 h, a challenge with *Escherichia coli* caused neutrophils to have impaired phagocytic activity, with higher bacterial colony counts in the lungs of these animals, possibly because of impairment in neutrophil function under sustained hypercapnic acidotic environment [17]. Hypercapnia worsened injury and induce apoptosis in alveolar type 2 epithelial cells via a nitric oxide-dependent pathway in an in vitro model with fetal rat alveolar cells pre-incubated with lipopolysaccharide (LPS) and cytokines [18]. Also, hypercapnia dose-dependently impaired alveolar cell proliferation and delayed wound repair in an in vitro scratch wound model of three different types of human lung cells [8]. These effects persisted with buffering of the hypercapnic acidosis. In line with this observation, in ex vivo and in vitro rat models of ventilator-induced lung injury (VILI), hypercapnic acidosis impaired membrane wound resealing. Carbon dioxide rather than pH reduced the rate of wound closure (cell migration) in a dose-dependent manner via NF-kB pathway inhibition. Furthermore, hypercapnia caused mitochondrial dysfunction and impaired cell proliferation in an in vitro model of cultured human alveolar epithelial cells by induction of miR-183, a microRNA, which in turn downregulated isocitrate dehydrogenase 2, a key enzyme of the tricarboxylic acid cycle that catalyzes the conversion of isocitrate to α-ketoglutarate, leading to mitochondrial dysfunction and impaired proliferation of alveolar epithelial cells [19].

### Effects of hypercapnia on humoral processes (Table 2)

Hypercapnia has been found to attenuate cytokine production and oxygen free radical formation in mice subjected to alveolar stretch [5, 15]. Furthermore, hypercapnia markedly reduced apoptosis, oxidative stress, and inflammation in alveolar epithelial cells from high-pressure ventilation-stimulated rat lungs [20]. Hypercapnic acidosis (HCA) exerts anti-inflammatory effects in rabbits with endotoxin-induced lung injury [16].

**Table 2:** Humoral effects of hypercapnia: summary of in vivo and ex vivo experiments on the effects of hypercapnia

Study	Experimental model	Applied CO_2_	Humoral effects
Shibata et al. [7]	Free-radical ex vivo (rabbit)	25%	HCA attenuated free-radical injury via inhibition of endogenous xanthine oxidase
Laffey et al. [6]	Pulmonary IR ex vivo (rabbit)	12%	HCA reduced TNF-α, 8-isoprostane, nitrotyrosine generation in the lung tissue and reduced apoptosis
Yang et al. [20]	VILI in vivo (rat) and in vitro alveolar epithelial cells	PaCO_2_ target 80–100 mmHg	HCA reduced caspase-3 activation (apoptosis), MPO, MDA, via ASK-1-JNK/p38 pathway inhibition
Otulakowski et al. [15]	VILI ex vivo (mouse) and in vitro alveolar epithelial cells	12%	Hypercapnia prevented activation of EGFR and p44/42 MAPK pathway in vitro. TNFR shedding (on ADAM-17 ligand induced by stretch injury) was reduced in vivo
Takeshita et al. [16]	Endotoxin in vitro pulmonary endothelial cells	10%	Hypercapnia reduced cell injury and prevented IκB degradation. NF-κB-dependent cytokine production was reduced
O’Toole et al. [8]	In vitro three cell respiratory lines	10, 15%	Hypercapnia inhibited p65 translocation and IκB degradation
Cummins et al. [13]	Endotoxin stimulated. In vitro six different cell lines	5, 10%	CO_2_ reduced the expression of innate immune effectors IL-6 and TNF-α
Wang et al. [21]	Endotoxin stimulation. In vitro human and mouse macrophages	5, 9, 12.5, 20%	Hypercapnia reduced cytokine release (IL-6, TNF-α)

*HCA* hypercapnic acidosis, *ADAM-17* ADAM metallopeptidase 17, *ASK-1* apoptosis signal-regulating kinase-1, *EGRF* epidermal growth factor receptor, *IkB* inhibitory kappa B, *IL-6* interleukin-6, *IR* ischemia-reperfusion, *JNK* c-Jun N-terminal kinase, *MDA* malondialdehyde, *MPO* myeloperoxidase, *NF-kB* nuclear factor kappa B, *p44/42 MAPK* p44/p42 mitogen-activated protein kinase, *TNF-α* tumor necrosis factor-α, *TNFR* tumor necrosis factor receptor, *VILI* ventilator-induced lung injury

On the contrary, hypercapnia has been found to selectively inhibit the expressions of proinflammatory cytokines in human and mouse macrophage cell lines [21]. In in vitro human cells’ experiments, hypercapnia inhibited activation of the NF-κB pathway [13, 22], independent of pH.

### Effects of hypercapnia on alveolar fluid clearance

Hypercapnic acidosis reduced alveolar edema formation by inhibiting an increase in pulmonary capillary included by free radicals [7], ischemia-reperfusion [14], and high-stretch ventilation in in vitro and in vivo models [23].

However, hypercapnia has also been found to decrease alveolar fluid clearance by decreasing Na^+^, K^+^-ATPase activity in the alveoli basal membrane in in vivo studies in large and small animal models [9, 24], in ex vivo studies using a rodent lung model [10], and in human alveolar epithelial cells [11].

## Physiologic effects of hypercapnia—animal and human studies

Several studies have shown various effects of hypercapnia on the respiratory system.

### Effects of hypercapnia on airway resistance

Hypercapnia may either decrease or increase airway resistance. Alveolar hypercapnia decreases airway resistance by relaxing smooth muscle small bronchi (a direct effect) in healthy subjects [25], while systemic hypercapnia causes vagal nerve-mediated constriction of the larger airway (an indirect effect) in animal models [26]. These opposing but balanced effects may produce a little net alteration in airway resistance [27].

### Effects of hypercapnia on the oxygenating capacity of the lung

Hypercapnia potentiates hypoxic pulmonary vasoconstriction with a reduction in intrapulmonary shunt, improving gas exchange in normal [28–30] and diseased lungs [31, 32]. Hypercapnia also increases lung compliance, directing ventilation to the underventilated lung regions resulting in a better match between ventilation and perfusion in the lung of dogs [33]. The mechanism might be through increased alveolar surfactant secretion and more effective surface tension-lowering properties of surfactant under acidic conditions [31]. However, in an in vivo rat model of prolonged *E. coli* pneumonia, hypercapnia lowered static lung compliance compared to normocapnia [17]. Sustained hypercapnia may impair neutrophil function, increasing bacterial load, contributing to increased lung injury and worst compliance [14].

Both hypercapnia and hypercapnic acidosis shift the hemoglobin–oxygen dissociation curve to the right and may increase hematocrit, augmenting the release of oxygen to the tissues in a canine model [34]. It also increases cardiac output through sympathoadrenal mechanisms [35]. The secondary rise in cardiac output is associated with increased preload, afterload, contractility, and elevated heart rate [36]. The overall effect is better oxygenation and improved global oxygen supply.

On the other hand, hypercapnia lowers alveolar oxygen tension by the following formula P_A_O_2_ = FiO_2_ (*P*_*B*_-47)—(PaCO_2_/*R*), where P_A_O_2_ is the alveolar oxygen tension, *P*_*B*_ is the barometric pressure, and *R* is the respiratory quotient. However, compared to alterations in FiO_2_ [37], the effect on alveolar oxygenation by alterations in alveolar ventilation is small.

The overall effect of these mechanisms is that blood oxygenation may remain mostly unchanged or improved.

### Effects of hypercapnia on diaphragm function

Hypercapnic acidosis preserves diaphragmatic contractility and prevents ventilation-induced diaphragmatic myosin loss and inflammation in pigs [38]. However, hypercapnic acidosis impairs diaphragmatic function in spontaneously breathing patients, through effects on afferent transmission by the vagal nerve [39].

The clinical effect of hypercapnia upon diaphragmatic function needs to be further elucidated.

### Effects of hypercapnia on pulmonary vasculature

In models of pulmonary hypertension induced by chronic hypoxia and right ventricular dysfunction, hypercapnic acidosis attenuates pulmonary hypertension, normalizes right ventricular function, and preserves the endothelial integrity and pulmonary vascular remodeling [40–42]. The effects of hypercapnic acidosis on vasoconstriction and resistance in the pulmonary circulation have also been found in humans [35]. These effects are exacerbated in the setting of preexisting pulmonary hypertension, such as found with ARDS [43].

## The impact of hypercapnia in acute respiratory distress syndrome

Supportive care with mechanical ventilation remains the mainstay of ARDS management with a goal to minimize lung injury caused by the forces created by mechanical ventilation. Treatment algorithms for ARDS typically begin with optimization of the settings to achieve the so-called lung-protective ventilation and proceed through increasing invasiveness based on physiological goals of gas exchange. These include higher positive end-expiratory pressures, lung recruitment manoeuvers, prone positioning, and extracorporeal removal of carbon dioxide.

### Permissive hypercapnia

Lung-protective ventilation with volumes limited to 6–8 mL/kg of predicted body weight (PBW) or lower, and plateau pressure < 30 cm H_2_O, dramatically increases survival [2]. As a consequence of reducing alveolar ventilation with lower tidal volumes, hypercapnia results. Recognizing that low tidal volume ventilation confers survival benefits by reducing lung stretch and the cyclical collapse of alveoli, clinicians have accepted hypercapnia, giving rise to the concept of permissive hypercapnia [44]. However, it remains unclear whether hypercapnic acidosis carries survival benefits independent of using low tidal volumes (Table 3).

**Table 3:** Randomized controlled studies in lung-protective ventilation and PaCO_2_ levels

Study	Mortality benefit	PaCO_2_ in control arm (mmHg ± SD)	PaCO_2_ in LPV arm (mmHg ± SD)	Buffer used
ARDSNet [2]	Yes	35.8 ± 8.0	40.0 ± 10.0	Yes
Amato et al. [66]	Yes	36.0 ± 1.5	58.0 ± 3.0	No
Brochard et al. [46]	No	41.0 ± 7.5	59.5 ± 19.0	No
Brower et al. [67]	No	40.1 ± 1.6	50.3 ± 3.5	Yes
Stewart et al. [68]	No	46.1 ± 10	54.5 ± 15	No

*LPV* lung-protective ventilation

In a retrospective analysis of the ARDS network, hypercapnic acidosis was associated with lower mortality in the group of patients receiving tidal volumes of 12 mL/kg PBW. However, there was no survival benefit in patients ventilated at tidal volumes of 6 mL/kg PBW [45]. It was hypothesized that lung-protective ventilation reduced lung injury caused by the ventilator to a point where the protective effect of hypercapnic acidosis could not be detected.

In a multicenter randomized clinical trial comparing low (7 mL/kg PBW) with conventional tidal volumes (10 mL/kg PBW), a trend towards higher mortality was observed in patients who developed hypercapnia and acidosis [46]. These findings resulted in a premature stop of that trial, making interpretation of the results difficult.

Recently, a post hoc analysis of three prospective non-interventional international studies in ARDS patients was published [47]. In this analysis, severe hypercapnia (PaCO_2_ > 50 mmHg) was associated with higher mortality and more organ failures compared to patients with normocapnia. Acidosis or the combination of hypercapnia and acidosis independently increased the risk of mortality in the intensive care unit. The incidence of severe hypercapnia increased significantly with the time (1998, 2004, and 2010) as a consequence of the diverse respiratory strategies practiced over the years, which may reflect the feeling of many intensivists that hypercapnia could be beneficial [47].

Finally, one retrospective analysis including over 250,000 ARDS patients receiving mechanical ventilation showed that patients who developed hypercapnic acidosis (pH < 7.35 PaCO_2_ > 65 mmHg) during the first 24 h of mechanical ventilation had significantly higher mortality than those who had compensated hypercapnia or normocapnia [48].

### Lung overdistension

The rationale for using positive end-expiratory pressure (PEEP) is to mitigate the injurious effects of repetitive opening and closing of lung units by restoring the functional size of the lung, promoting lung protection, and improving gas exchange and lung mechanics. However, high levels of PEEP may lead to overdistension of lung units, especially those that remained normally aerated within heterogeneous ARDS lungs, and this may increase alveolar dead space [49–51]. The consequence of this will be a rise of carbon dioxide levels.

In a recent international randomized clinical trial in ARDS patients, the use of the “open lung approach” actually increased 28-day mortality [52]. Notably, this trial used aggressive recruitment manoeuvers and “super high” levels of positive end-expiratory pressure compared to previous trials [53–55]. It is also worth to note that patients in this trial had much higher blood carbon dioxide levels than patients in previous trials that tested the “open lung approach.” It could be hypothesized that this reflects an increase in dead space secondary to overdistension.

## Extracorporeal CO_2_ removal (Table 4 and Additional file 1)

Despite lung-protective ventilation strategies, up to 30% of patients with ARDS have evidence of tidal hyperinflation, representing a potential risk of VILI [56]. A strategy of “ultraprotective ventilation” with tidal volumes lower than 6 mL/kg PBW requires extracorporeal decapneization.

**Table 4:** Relevant clinical studies of ECCO_2_R in ARDS patients

Study	ECCO_2_R technique	Description and results
Terragni et al. [56]	Modified continuous VV hemofiltration system with membrane lung via a 14-Fr single dual lumen catheter (femoral) with an extracorporeal blood flow of 191–422 mL/min	Prospective study. Ten ARDS patients with 28 ≤ Pplat ≤ 30 after 72 h of ARDSNet ventilation were placed on ECCO_2_R and had a progressive reduction in V_T._ V_T_ was reduced from 6.3 ± 0.2 to 4.2 ± 0.3 mL/kg PBW and Pplat decreased from 29.1 ± 1.2 to 25.0 ± 1.2 cmH_2_O (*P* < 0.001). Consequent respiratory acidosis was managed by ECCO_2_R. Improvement of morphological markers of lung protection and reduction in pulmonary cytokines (*P* < 0.01) after 72 h of ventilation with *V*T < 6 mL/kg PBW. No patient-related complications. Membrane clotting in three patients.
Bein et al. [58]	Femoral AV pumpless extracorporeal lung assist (PECLA) via a 15-Fr arterial cannula and 17-Fr venous cannula with a mean extracorporeal blood flow of 1.3 L/min	Randomized controlled trial. Moderate/severe ARDS after 24-h stabilization period with higher PEEP. Randomized to ECCO_2_R group with ~ 3 mL/kg PBW ventilation or control group with ~ 6 mL/kg PBW ventilation. There were no significant differences in VFDs at day 28 (10 vs. 9 days, *P* = 0.78) or day 60 (33 vs. 29, *P* = 0.47). Post hoc analysis showed that patients with P/F ≤ 150 at randomization in ECCO_2_R group had a significantly shorter duration of ventilation (VFDs at day 60, 41 vs. 28, *P* = 0.033). Significantly higher red blood cell transfusion in the PECLA group up to day 10 (3.7 vs. 1.5 units, *P* < 0.05).
Fanelli et al. [59]	VV configuration via a 15.5-Fr single dual lumen catheter (femoral or jugular) with a mean extracorporeal blood flow of 435 mL/min	Prospective study. Moderate/severe ARDS. V_T_ was reduced from baseline to 4 mL/kg PBW. Low-flow ECCO_2_R was initiated when pH < 7.25 and PaCO_2_ > 60 mmHg. ECCO_2_R was effective in correcting pH and PaCO_2_. Life-threatening hypoxemia such as prone position and ECMO were necessary in four and two patients, respectively.
Schmidt et al. [69]	VV configuration managed with renal replacement platform via a 15.5-Fr single dual lumen catheter (femoral or jugular) with a mean extracorporeal blood flow of 421 mL/min	Prospective multicenter study. Twenty-two patients with mild/moderate ARDS V_T_ gradually reduced following 2-h run-in time from 6 to 5, 4.5, and 4 mL/kg every 30 min and PEEP adjusted to reach 23 ≤ P_plat_ ≤ 25 cmH_2_O. No patients required ECMO. No worsening oxygenation. Low-flow ECCO_2_R managed by RRT platform easily and safely enabled ultraprotective ventilation. Performance of RRT ECCO_2_R in severe ARDS patients not known.
Combes et al. (NCT 02282657) [70]	VV configuration 15.5 to 19 Fr single dual lumen catheter (femoral or jugular) with three different devices.	Prospective multicenter study. Ninety-five patients with moderate ARDS. V_T_ progressively decreased to 4 mL/kg PBW. PEEP adjusted to reach 23 ≤ Pplat ≤ 25 cmH_2_O. Objective to maintain PaCO_2_ ± 20% of baseline values obtained at V_T_ 6 mL/kg IBW with pH > 7.30. ECCO_2_R was able to reduce Pplat from 26 ± 5 cmH_2_O to 23 ± 3 cmH_2_O (*P* < 0.01) in 73% of patients. ECCO_2_R was able to increase PEEP from 12 ± 4 cmH_2_O to 14 ± 4 cmH_2_O (*P* < 0.01). ECCO_2_R allowed ∆P reduction from 13 ± 5 to 9 ± 4 cmH_2_O (*P* < 0.01). There were no significant changes in pH, PaCO_2_, and PaO_2_/FiO_2_ with V_T_ reduction to 4 mL/kg/IBW ECCO_2_R device length: 5 (3–8 days). Derecruitment/hypoxia (*n* = 2) that need to increase V_T_, hemolysis (*n* = 3). Hemorrhage at the cannula insertion point (*n* = 4), pneumothorax (*n* = 1).

This approach has been tested in two trials in ARDS patients, resulting in less lung injury caused by ventilation [57] and a decrease in the number of ventilation days [58]. In one feasibility study, ECCO_2_R facilitated ventilation with ultralow tidal volumes near to 3 mL/kg PBW, while preventing hypercapnic acidosis [59]. These findings were confirmed in a recently completed international multicenter randomized clinical trial (ClinicalTrials.gov/ct2/show/NCT02282657). One currently recruiting randomized clinical trial evaluates whether ultraprotective ventilation by employing ECCO_2_R affects 90-day mortality in patients with hypoxemic acute respiratory failure (ClinicalTrials.gov/ct2/show/NCT02654327).

Although ECCO_2_R seems to be effective in mitigating hypercapnic acidosis and possibly in reducing VILI and mechanical ventilation days, ECCO_2_R may have pulmonary and hemodynamic consequences. It can be associated to adverse effects such worsening hypoxemia and increased FiO_2_ requirements due to a decrease in mean airway pressure, low ventilation-perfusion ratio, and lower partial pressure of alveolar oxygen secondary to a decreased lung respiratory quotient [60, 61]. Besides, because of the low flow system of ECCO_2_R, higher anticoagulation requirements are needed in order to maintain ECCO_2_R efficiency and performance. Therefore, significant complications may occur as a consequence of anticoagulation or catheter insertion with hemodynamic instability and a higher number of red blood cell transfusions needed [59, 62–64].

It is worth to say that ECCO_2_R may be a promising adjuvant therapeutic strategy for reducing the impact of mechanical ventilation through ultraprotective ventilation rather than to mitigate hypercapnia in patients under conventional lung-protective ventilation. For the time being, the available literature does not provide definitive information on the usefulness of ECCO_2_R in patients with acute respiratory failure. Its use for the moment is experimental.

## Conclusion

It is increasingly recognized that CO_2_ is much more than just a waste product of cellular metabolism. Indeed, it should be seen as a potent biological agent that exerts protective as well as harmful effects. Hypercapnia may develop in ARDS patients, and what the exact impact of high carbon dioxide levels on the outcome remains uncertain. More importantly, whether it should be accepted or whether it should be prevented or treated with invasive techniques for extracorporeal removal remains highly uncertain.

## Additional file

Additional file 1: ** Table S1.** Ongoing studies of ECCO_2_R in ARDS. (DOCX 16 kb)

## Abbreviations

ARDS: Acute respiratory distress syndrome
CO_2_: Carbon dioxide
ECCO_2_R: Extracorporeal carbon dioxide removal
HCA: Hypercapnic acidosis
LPS: Lipopolysaccharide
NF-kB: Nuclear factor kappa B
PaCO_2_: Partial arterial pressure of carbon dioxide
PaO_2_: Partial alveolar pressure of oxygen
PBW: Predicted body weight
VILI: Ventilator-induced lung injury
