# Supplementary material for: The role of hypercapnia in acute respiratory failure
Source: Intensive Care Med Exp. 2019 Jul 25;7(Suppl 1):39. doi: 10.1186/s40635-019-0239-0 (PMC6658637; doi:10.1186/s40635-019-0239-0)
Supplement: Supplementary file 1 — Table S1. Ongoing studies of ECCO2R in ARDS. (DOCX 16 kb) [file 40635_2019_239_MOESM1_ESM.docx]

**Additional file 1: Table S1: Ongoing studies of ECCO_2_R in ARDS**

| ClinicalTrials.gov Identifier/Official Title | Study design | Primary outcome | Inclusion criteria | Estimated enrollment | Status |
| --- | --- | --- | --- | --- | --- |
| NCT03525691. Enhanced Lung Protective Ventilation With ECCO_2_R During ARDS (PROVE) | Randomized single center control trial | 20% decrease in PaCO_2_ after initiation of ECCO_2_R at V_T_ of 4 mL/kg PBW as compared to 4 mL/kg without ECCO_2_R | Moderate to severe ARDS with onset < 48 hours and driving pressure ≥ 11 cmH_2_O | 14 patients | Recruiting |
| NCT033303807. Correction by ECCO_2_R of Hypercapnia in Patients with DVP in Moderate to Severe ARDS Under Protective Ventilation (COVAP) | Prospective multicenter regional study | Correction of hypercapnia within 2 hours after ECCO_2_R initiation | Moderate to severe ARDS  Pulmonary vascular dysfunction (PVD)  Refractory hypercapnia (PaCO_2_ ≥ 48 mmHg in spite of the reduction of the instrumental dead space and the increase in respiratory rate | 20 patients | Not yet recruiting |
| NCT02252094. Ultra-protective Pulmonary Ventilation Supported by Low Flow ECCO_2_R for Severe ARDS (U-Protect) | Randomized control trial | Ability of ECCO_2_R to achieve a plateau pressure of ≤ 25 cmH_2_O | ARDS with PaO_2_/FiO_2_ ≤200 for > 6 hours with FiO_2_≥0.5 and expected to require mechanical ventilation for > 48 hours | 50 patients | Recruiting |
| Registry on the Experience of Extracorporeal CO_2_ Removal in Intensive Care Units (REXECOR) | Observational | Incidence of ECCO_2_R use | Patients implanted with ECCO_2_R in acute exacerbation COPD and ARDS | 200 patients | Recruiting |
| NCT02654327. pRotective vEntilation With Veno-venouS Lung assist in Respiratory Failure (REST) | Randomized multicenter control trial | Mortality at 90 days | Acute and potentially reversible cause of acute respiratory failure with invasive mechanical ventilation within 48 hours of onset of hypoxemia (PaO_2_/FiO_2_ ≤ 150) | 1120 patients | Recruiting |
